# Key factors for child participation – an empowerment model for active inclusion in participatory processes

**DOI:** 10.3389/fpsyg.2023.1247483

**Published:** 2023-10-06

**Authors:** Rita Karoline Olsen

**Affiliations:** Department of Education and Lifelong Learning, Norwegian University of Science and Technology, Trondheim, Norway

**Keywords:** adult-child interactions, child participation, children’s agency, empowerment, methodological strategy

## Abstract

Child participation is advocated for in many contexts related to children, such as in education and health services, but also in everyday life settings. To facilitate children’s real opportunities for participation, it is proposed that children need to be adequately empowered to liberate the child’s autonomous voice and activate their powers of action. This involves an understanding of children as opinion-forming and social individuals, who play an active part in social relationships. This article aims at a theoretical exploration of empowerment in the organization of ethically justifiable participation situations with children. With a semantic view of theory, an eclectic research design has been used to search for a theoretical framework based on basic human needs to be an active agent in one’s own life. The purpose is to define key factors that promote the sense of empowerment as a prerequisite for being an active participant in interaction with adults. This conceptualization aims to raise clearer guidelines for the implementation of child participation and presents an empowerment model with four dimensions for the active inclusion of children in participatory processes with adults. These dimensions are information, autonomy, recognition, and alliance.

## Introduction

Children in formal and administrative procedures [United Nations Convention on the Rights of the Child (UNCRC), 1989] can be seen as a group that is in a particularly vulnerable situation. Inclusion often takes place in an adult-constructed context on the adults’ initiative and premises, and the procedures can be confusing and incomprehensible. It is understandable that the children still want to be involved and participate in the decisions that concern them and affect their situation, and they have proven capable of doing so, even with complicated questions (Sinclair et al., 2002; Leeson, 2007; Woolfson et al., 2010; Van Bijleveld et al., 2015). The experience of powerlessness is a risk element for children’s development, especially in situations where they are dependent on professionals (for example, in treatment, investigation measures, legal proceedings, and formal processes – see the Convention on the Rights of the Child, art. 12, paragraph 2). Participation and active involvement are a counterweight to this feeling, where children should be able to take part in their own matters and decisions that concern them, and experience being heard and taken seriously. Child participation is also highly relevant in relation to everyday life situations. Good inclusive participation processes, where children are given space to be an active agent in shaping their own lives and the society they live in, are thus important for participation to be realized. Child participation has nevertheless proven difficult to implement in practice, and several agencies have highlighted the need for more knowledge and tools to facilitate good participation processes (Archard and Skivenes, 2009; Graham and Fitzgerald, 2010; Vis et al., 2020; Ulset et al., 2023). Several investigations have shown that participation works at a minimum where services and professional fields lack both clarity in what participation is and a conscious strategy for how this should be carried out (Shier, 2001; Children’s Ombudsman, 2017; NOU 2020:14, 2020). The purpose of this paper is to present a theoretical model for the active inclusion of children in participatory processes and to contribute to a knowledge foundation for facilitating ethically sound participation practices. The ethical basis is built on an understanding of the adult’ responsibility to facilitate children’s opportunity to formulate their wishes, needs and views, and then to be able to express these as a prerequisite for active participation. This is to prevent participation taking place on a tokenistic level. The model is constructed through an eclectic exploratory conceptualization process based on consideration of the skewed power relations between adults and children in adult-constructed contexts and supports a modern development psychologic perspective to child participation, which emphasizes promoting the child’s agency and resilience as fundamental for being able to promote real participation.

An understanding of Blumer’s (1954) sensitizing concepts has guided the theoretical approach and allows the researcher to start with a concept to give direction and develop theoretical/analytical tools (Flemmen, 2017). Research approach through sensitizing concept is a sociological tool to facilitate a position where one can perceive something new, starting from background ideas that inform the overall research question (Bowen, 2019). The basis for this study has been the problematization and ethical consideration of the child’s vulnerable position when facing adults in judicial and administrative proceedings, where the child is expected to perform in an unfamiliar context by actively participating, making up their own minds about a case, and presenting their views.

## Construction of a conceptualization model for child participation

The theoretical model of child participation is discussed in this article mainly in the context of formal meetings between children and adults in professional roles. Through a semantic theory approach, it is nevertheless argued that the model can be generalized to a greater extent and applied flexibly in various situations where the goal is for children to take an active participant role in collaborative relationships with adults. The approach is taken from, p. a., Alvesson and Sköldberg’s (2009) arguments that reality consists of underlying patterns and structures, and Van Fraassen’s (1980) philosophy that the scientific goal is to produce theories that are empirically probable and sufficient. The participation model presented here is based on theories about basic mechanisms to meet a human need to be an active agent in one’s own life. Through a selection of basic parameters for representing the complexity of the phenomenon of child participation, independent casual factors will be assessed together and create a simplified and abstract model of the phenomenon. In this work, inspiration has been drawn from Suppe (1989, 2000), who highlights the model as the most important source of scientific knowledge. The model should therefore not describe the phenomenon of child participation in detail, but rather as scenarios that describe the system of the phenomenon, as if it stood outside its complex context.

The theory is thus intended to cover several individual cases, not just one and within a given context. The parameters that have been selected and presented here are those that are considered significant to the relevant situations for child participation. Therefore, the parameters that are context-dependent and situation-dependent have been outsourced. This contributes to make the theory more flexible and applicable, but also means that the theory must be used with a reflected awareness of what the theory means. This can be challenging when the theory is to be applied in practice (Kvernbekk, 2002), but will also be discussed in the light of practical examples throughout the paper. The theory is concentrated around a methodological idea for empowerment as a methodological approach to the sense of participation in formal meetings between adults and children. The model’s selected empowerment parameters aim to activate the child’s agency in encounters with adults in participation situations. The methodological idea does not present concrete methodological procedures and strategies but emphasizes some basic principles that are common to all individual cases. This is about a basic understanding of participation as a relationally anchored mechanism. The flexibility of the model therefore lies in the need to experience that participation is defined regardless of age, person, ability, expertise or situation. The conceptual model is built on principles of basic needs that all people have, both to experience the world as predictable and meaningful (Antonovsky, 1987), the need for autonomy, competence and relatedness (Deci and Ryan, 1985; Deci and Ryan, 2000), the need to feeling recognized and confirmed through interaction with others (Hegel, 1999; Schibbye, 2009), as well as the experience of a collaborative alliance between the adult and the child characterized by positive interdependence (Bordin, 1979). The theories linked to the model describe the behavior of a population of idealized individuals, whose behavior is only a function of situations of involvement between adults and children. According to Suppe (1989), this means that scientific theories, such as the one here relating to child participation, treat the phenomenon as if it only consisted of, or could be characterized by, the selected parameters. The behavior of real people can also be characterized by many other parameters that fall outside the theory.

## An empowerment model for active inclusion in participation processes

**Figure 1:**
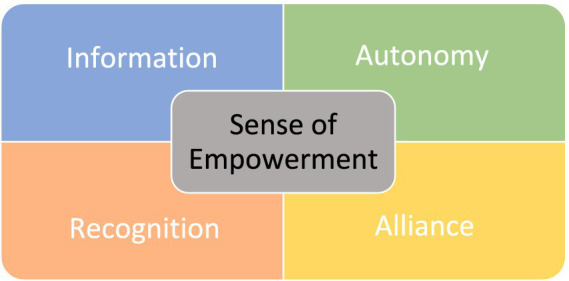
An empowerment model for the active inclusion of children in participatory processes.

The understanding of child participation as it relates to legal and administrative procedures is here about actively facilitating the inclusion of the child in the participation process, facilitating the experience of agency and autonomy, and giving them real opportunities to participate on their own terms. This approach places emphasis on supporting the child to activate their own agency, and a theoretical model with four dimensions based on this understanding of empowerment is proposed in the further work. The four dimensions of facilitating children’s sense of real opportunity for participation are the following: **A: Information** – experience of having an overview and control over the situation so that one has the prerequisites to be able to reflect, shape one’s own views, and make choices; **B: Autonomy** – experience of being able to freely express one’s thoughts, reflections, and opinions; **C: Recognition** – the child’s experience of being heard and taken seriously through support and acceptance from the adult; and **D: Alliance** – sense of self-worth and being an important factor in the collaborative relationship with an influence on the work process. Proposals for a visual presentation of these four dimensions are presented in Figure 1.

## The understanding of child participation

For further exploration of children’s participation as a concept, it is useful to reflect on leading discussions on the topic. Child participation is both a legal right, through the Convention on the Rights of the Child and national guidelines [United Nations Convention on the Rights of the Child (UNCRC), 1989], and a view of values and an understanding of children as equal human beings and citizens (Bae, 2010; Lúcio and I’Anson, 2015). Since the 1960s, a wide range of participation models have been published to describe different forms and levels of participation, from manipulation and tokenism on the one hand to situations where children have both initiated and lead the processes on the other (for example, Hart, 1992; Treseder, 1997; Shier, 2001; Lundy, 2007). Different methodological approaches are also presented, like in research where children are often active in processes involving information gathering either from themselves or from others (Clark and Moss, 2001; Kellett, 2011). There has also been attention to the actual value of child participation as a benefit in service development and research and as an element of facilitation for the individual child (Askheim, 2017; Skauge et al., 2021). Participation is also known to have an empowering value (often related to the field of substance abuse and mental health), where the participation has a therapeutic effect on the participants, generating self-confidence and a belief in one’s own mastery. These approaches to child participation refer to different activities and influence (outcomes) of participation. Few of these however, address the issue surrounding the child’s own experience of facing these contexts and the child’s prerequisites for adopting an active role in these situations.

Emphasis on the child’s experience of participation is about setting the conditions correctly so that the child, despite the skewed power relationship with the adult, experiences real and meaningful participation in these processes. This will be about individual and relational factors that build up the child’s experience of being able to be an independent and autonomous individual and to act based on their own wishes and needs. This can be linked to empowerment (Rappaport, 1981) as a protective developmental element, and it neutralizes factors that make participation happen on a superficial and tokenistic level. Empowerment is seen as a decisive factor for people’s development of good mental health and quality of life (Prilleltensky, 2010; Szoko et al., 2022) and has become an important argument for child participation in matters that concern them (Thomas, 2007; Wong et al., 2010). Treseder (1997) argues that there should be no limits to the involvement of children and young people, but they will not be able to engage in child-initiated and directed projects right away and need to be empowered adequately to be able to fully participate. This approach to child participation considers some basic ethical issues related to the child’s sense of participation in the process and the child’s actual contribution in these.

Prerequisites for active participation in participatory situations thus entail (a) reducing (considering) the barriers that prevent the individual’s autonomous self from being expressed (often this is about power structures and power inequalities) and (b) highlighting the individual resources and viewpoints, which is in line with the aim and purpose of the participation processes. The issue that arises when facilitating the implementation of child participation concerns how to understand child participation as an active inclusion in collaborative processes. With the respect to this issue, power structures and power inequalities between adults and children emerge as central, as well as the child’s sense of empowerment in the face of the adult constructed context. In the following text, and through a theoretical exploratory approach to child participation as a relational concept, prerequisites for children’s active participation and autonomous voice are discussed.

## Child participation as an ethical practice

Quality assurance of child participation involves an awareness of facilitating the children’s autonomous voice and real participation, whether the situation is really in the child’s best interests, and whether those who invite (engage in) participation know how to handle the complexity of appropriate, meaningful, and authentic participation for children (Viviers and Lombard, 2013). Some of the ethical assessments in such processes are about ensuring that the children can feel freedom of choice linked to their participation, experience being able to make real choices, and that their contribution to the work is important and significant (Olsen et al., 2022). It is also important that the participation situation itself does not produce an experience of powerlessness, especially if the children are already in a vulnerable situation. The aim and purpose of the participation (the matter) must be clearly communicated (Olsen, 2022), and the participants should be actively involved in all phases (Hagquist and Starrin, 1997). Smith et al. (2002) also find this when they have explored international and continental human rights, and the influence of adult–child relationships, power, and status on children’s right to participate and the concept of ethics in child participation. Save the Children USA (2004, p. 6) indicates that an ethical approach to child participation is about ensuring that children can freely express their views and opinions, that they are listened to, and that their contribution is taken seriously and given due weight. This requires that account be taken of the inevitable skewed distribution of power between adults and children. Viviers (2010) has identified a number of factors that are important for the ethical facilitation of child participation: the adult’s capacity to understand and facilitate meaningful participation, openness in working with the children, the children’s right to self-determination, available information and mutual communication, respect for the children’s views and recognition of their ability to contribute, emphasis on the children’s safety, inclusion and respect for differences, adequate resources, and assessment of the child’s best interests (see also Thomas and O’Kane, 1998; Save the Children USA, 2004; Ergler, 2017).

In an Australian study, Graham and Fitzgerald (2008) investigated children’s views on participation. The findings in this study pointed to the importance of being seen and recognized for who they were here and now through being included and taken seriously. This led to increased self-confidence and self-respect. Children have also in other studies strongly expressed the importance of having a say in decisions that were made about them (Kilkelly et al., 2005), and they have been concerned with being accorded dignity and respect and being able to contribute to discussions (Morrow, 1999). Wyness (2006) argues for creating a child-friendly space that reflects the local needs, the children’s interests, and their preferred way of being included so that the children’s voices do not become a reinforcement of the adults’ control. The children themselves describe how these spaces for activity are formed through receiving customized information, enough time to think about the various issues on which they are supposed to have an opinion, a child-led infrastructure to formulate their views, and training for adults in how they can overcome resistance to the child’s voice (Bennett Woodhouse, 2003).

In a study from 2007, Lundy has conceptualized Article 12 of the Convention on the Rights of the Child as an attempt to embrace holistic thinking around the intention of the concept of “children’s voice.” She presents four elements in rational chronological order, where the first two, *space* and *voice*, deal with children’s right to express themselves, while the last two, *audience* and *influence*, deal with the child’s right to be heard and influence decision-making. This model has been influential in several places in the professional community [e.g., as part of Ireland’s national strategy on children and young people’s participation in decision-making 2015–2020 – Department of Children and Youth Affairs (DCYA), 2015] and is also linked to a checklist for participation, which aims to help organizations that work with and for children and young people to comply with Article 12 of the UNCRC [Department of Children and Youth Affairs (DCYA), 2015]. These four parts are about *space* – children’s opportunity to express their opinions, *voice* – children must be assisted to be able to express their opinions, *audience* – the children’s views must be heard, and *influence* – the children’s views must be taken into account. These elements of participation highlight the adults’ responsibility to be facilitators and to ensure and encourage children to express their opinions in a safe space without fear of criticism (Lundy, 2007).

Child participation as a subject still lacks a holistic model of understanding, which highlights the interrelational process and emphasizes the dyadic cooperative relationship between adults and children in participation processes. This implies an understanding of child participation as a collaborative process between children and adults and that children can explore and develop their autonomous voice in the process. Such a model must therefore place emphasis on actively including the child in the process so that they are given the conditions to be active agents and work together with the adult in the process.

Unlike the traditional participation models, as well as the ongoing discourse about the extent to which children should participate in decision-making, this empowerment model of participation primarily addresses the adult’s responsibility to facilitate children’s prerequisites for active participation, as a counterweight to tokenistic practices and thus a part of a quality assurance of ethically sound practice based on the child’s own experience of participation in meetings with the adults. Reference can be made to a co-creation project by Olsen (2022) where children have been collaborative participants in the development of a participation tool called The Sense of Empowerment Inventory for Children (SEIC). In a collaborative approach in this project with 7 children, a meaningful assignment is described as the starting point for the participation process, and experience of value (autonomy), safe social environment (recognition), inclusion through information (information) and exploratory working method (collaborative alliance) are described as capabilities with the conceptual understanding of child participation.

Lundy and McEvoy (2012) also describe a similar methodological practice through what they call the Children’s Research Advisory Group (CRAG) where the goal is to assist capacity building to enable the children to form their views and participate on their conditions. This enabling and support to shape their own views is about strengthening the child’s experience of autonomy and self-determination and creates a space for exploration and reflection on one’s own views and opinions. Access to information is described as a key element for children to be able to form their view. This collaborative approach considers the children’s need for support in participation situations and makes room for a respectful practice between adults and children. Additionally, co-reflection is described leading to an increased experience of empowerment and thus increased active participation in the collaborative process.

Also Saglietti and Zucchermaglio (2022) discuss the child participation in everyday life practices such as mealtime situations and describe, among other things, how traditional teacher-pupil relationships are characterized by control and a top-down attitude, where children are socialized to listen to adults’ own voice, and to follow it, leaves little room for the child participation and agency. Saglietti and Zucchermaglio (2022) therefore argue for a greater degree of metacommunication with the children, characterized by open questions and a demand for an affective attitude over argumentative strategies. Such communication should ensure that the communication is comprehensible to all participants and has room for opinion negotiation and argumentation, children’s refusal, and alternative opinion-making. Problematization of the traditional teacher-student discipline is also raised in the study by Olsen et al. (2022) where children have been interviewed about their experience of being an active participant in collaborative processes together with adults. The findings in this study also promote the importance of a freedom to explore thoughts and ideas and express oneself independently of demands and expectations, a sense of significant contribution where one’s contribution is heard and taken seriously, and a safe social context characterized by openness, acceptance, and support.

## The child as agent – ability to take control of one’s own life

In modern childhood sociology, children’s agency is a key theme where socialization into self-regulation is one of the main elements in the modern understanding of childhood (Ochs and Izquierdo, 2009). The possibility to influence and change certain matters is seen as a central aspect of the development of agency. Aronsson et al. (2018) defines children’s agency as how children contribute to their own development through the way they meet demands and engage in activities. Although in developmental psychology there has been a focus on agency following the child’s maturity, in a more modern understanding agency is linked to environmental conditions and relationships between people. We therefore need to understand more of how children develop agency in relation to the social world of which they are a part of and explore how autonomy and interdependence can be analyzed as interrelated (Holzkamp, 2015; Aronsson et al., 2018 p. 175). Children develop their agency in interaction with other children and the social community. Children in difficult life situations may tend to keep caregivers or resource persons in their environment at a distance, which reduces their opportunity for support and the development of agency. Aronsson et al. (2018) therefore argues for the need to understand more about how professionals collectively work with conflicts, dilemmas and problematic themes embedded in the children’s difficult life situations in ways that support and expand their opportunities for agency.

People have a basic motivation to master their own lives, which means being able to influence and, to some extent, control the direction life takes. This can be related to human agency (Bandura, 1986), which is understood as people’s ability to take control of their own lives. The term does not only refer to a competence that people can develop, but it equally refers to opportunities in the environment in which people find themselves. In participation processes, this will be an important term to understand the importance of the child being given room both for action and the activation of their agent forces through collaboration with the adult. In Antonovsky’s (1987) theory of “sense of coherence” (SOC), he presents the importance of experiencing the situation one is faced with as understandable, manageable, and meaningful so that people can handle their lives and act upon challenging situations in which they find themselves. This is about the stimuli that come from the internal and external environment being structured, predictable, and recognizable and about having enough resources at one’s disposal to be able to cope with the demands (what is expected of one), which must be worth engaging in. The SOC must not be confused with the experience of control (in the context of participation as co-determination or responsibility), but this is about experiencing the world and what happens around you as predictable and understandable (Antonovsky, 1987). Instead of the experience of being able to solve or make decisions, as in participatory situations, rather creates the conditions for being able to adapt and take action to protect oneself, which reduces chaos and stress.

Self-determination (Deci and Ryan, 1985) is an important part of self-organization and is reflected in an experience of integrity, will, and vitality that follows self-regulated action (Ryan, 1993). When people experience themselves as self-determined, they see themselves as initiators of their own activities and actions together with having the ability to make their own choices (Deci and Ryan, 1985, 2000). This is an important function for being able to act independently and thus have agency linked to one’s own functioning and achievement, and it is a central part of being an active participant in one’s own situation (and problem-solving) in contrast to taking a passive role, in which the person often feels powerless. Through self-determination theory (Deci and Ryan, 1985), three basic needs – autonomy, competence, and relatedness – are promoted as governing people’s tendency to activate their strengths and resources. Self-determination is thus fundamental to one’s motivation, which means that freedom to engage is essential for self-directed action. The adult’s autonomy-supportive behavior can help to identify, support, and develop the children’s internal motivational resources (Deci and Ryan, 2000).

## Empowerment as a countermeasure to powerlessness

From a medical understanding, empowerment (Rappaport, 1981, 1984) stands as a countereffect to powerlessness, and the term refers to measures to increase the degree of autonomy and self-determination in people to enable them to promote their own interests in a responsible and self-determined manner and to act on their own. These will be important functions/conditions for being able to take an active role in situations requiring participation. This is a process of becoming stronger and gaining more self-confidence, especially when it comes to controlling one’s own life and asserting one’s rights. Sue (1981) suggests that the understanding that best expresses the need for empowerment is the relationship between the inner experience of control and the outer experience of responsibility. This way of understanding empowerment is limited to a “state of mind.” Based on social behavior and social learning theory, Bandura’s (1994a) concept of self-efficacy is often linked to empowerment. Self-efficacy refers to a person’s belief that they have the ability to succeed in a given situation. Empowerment from a self-efficacy perspective focuses on increasing perceived self-efficacy and movement toward positive health behaviors, sense of control and choice, as well as power through coping experiences, social modeling, social persuasion, and psychological responses (Bandura, 1994b). Prilleltensky (2001) distinguish between children as agents of values and children as recipients of values. As agents of values, children are seen as capable of and responsible for contributing to themselves and their family and society. Children are thus not seen as passive objects of internal and external influence but as active agents in their social world. The experience of empowerment in meeting others, especially in unequal power relations, will therefore play an important role in the sense of agency and mobilization of energy to work in participatory processes with adults.

In the collaborative approach to participation as activating the child’s agency, it is appropriate to turn to Rissel’s (1994) term of psychological empowerment. Differentiated from community empowerment, which is about the fair distribution of limited resources, psychological empowerment is an expanding phenomenon that does not come at the expense of the other’s empowerment (Hagquist and Starrin, 1997). Psychological empowerment refers more to a process that increases strength and self-confidence, especially when it comes to controlling one’s own life and asserting one’s rights. This is both a process of self-empowerment and of professional support of people, enabling them to overcome their sense of powerlessness and lack of influence and to recognize and use their sense of resources and opportunities. An important perspective on empowerment among children and young people in relation to adults is that this relationship can hardly be seen as equal. Heffner (1988) problematizes the understanding that well-intentioned adults can empower powerless young people and believes that this is not possible. Although empowerment is about self-activity, adults can contribute by creating frameworks and structuring experiences in such a way that they support young people in empowering themselves (Heffner, 1988). By creating supportive structures, one can thus promote an increased sense of empowerment (Rissel, 1994; Hagquist and Starrin, 1997). The aim of the empowerment model presented in this paper is thus to strengthen and develop individuals’ own abilities, which is a necessary process in the work to promote active agents in participation processes (Green et al., 1995). The basic idea for an empowerment model is that those involved actively participate in all phases of work for change and constantly maintain an active dialog with each other, in which the adults play an important role as facilitators of an empowerment process (Hagquist and Starrin, 1997).

## Autonomy and independence

Related to empowerment lies the concept of self-determination and autonomy as a component of agency, which deals with a person’s inner independence and ability to decide their own actions. The background for this term is voluntarism and the idea of free will. The theories come from Kant (1964) and are understood as our ability to manage our lives based on assessments of what is right and wrong, good and evil. This also implies an idea that dignity is linked to a respect for one’s own and others’ reason and will. The term is linked to an individualistic understanding that emphasizes the individual’s value and respect for the individual’s freedom of action, which is an important basis for democracy and human rights.

Schibbye (1988, 2009) has developed the theory of dialectical relational understanding, which emphasizes the appreciative relationships between people as a source of development and growth. The theory is inspired by Hegel (1770–1831), who claimed that humans need each other to develop into independent individuals. Dialectical relational theory is also based on Mead’s (1934) mirroring theory. Through relationships with others, recognition can be realized, and these relationships are characterized by equality (Schibbye, 2009). In a collaborative approach to participation, equality in the dialog will be a central component. The term recognition therefore includes acknowledging, respecting, and accepting. Hegel (1999) believed that in this way one develops self-awareness, explaining this in more detail through theories about the struggle for independence and the struggle for self-assertion. These are important components for the child’s ability to first have room to define their own wishes and needs related to the specific topic and issue and also to be able to express their own independent opinions that are detached from the adult’s expectations and views. An appreciative approach from the adult will therefore be able to create a sense of security that supports the child’s ability to promote their own cause, wishes, and needs. Listening, understanding, acceptance, tolerance, confirmation, and openness are ways of being that overlap with each other and are dialectical. Emphasis on autonomy directs the focus toward the first-person perspective and an understanding that the individual knows his needs best. From this stems the importance of listening to the needs and perspectives of others.

In discussions surrounding what true autonomy entails, Solberg (2020, 2022) problematizes the autonomy perspective as being too much about choice. When a person is competent to consent, information is often provided so that the person can make informed choices, and through a non-paternalistic freedom of action, participation is reduced to a private matter. As an alternative theoretical tradition, Solberg (2020, 2022) uses the concept of relational autonomy and emphasizes that people “become themselves” through relationships and not from being detached from relationships. We are fundamentally connected to other people, and our own choices are linked to values related to social values and our own position and role in the social community. Autonomy is therefore not only about enabling a person to make choices through adequate information, but the relational aspect of one’s place in the community is important for the liberation that occurs through the social community’s caring intervention. Solberg (2020, 2022) presents this understanding as a concept around “co-selection,” which reflects the importance of collaboration and community more than the concept of autonomy alone. Relational autonomy nevertheless becomes an important factor in the liberation/strengthening of the person’s self-worth and agency and as a tool for the person’s self-assertion (own values, needs, and wishes) in co-forming with the community. This forms the basis of a collaborative approach to participatory situations between adults and children. An understanding of co-selection is then about a partnership where both/all parts work together with a collective understanding of the aim of a common solution. Such a partnership requires that the adult takes an equal role in participation with the child and recognizes his responsibility to contribute his expertise and perspective to the situation. It is thus important that the child’s right to participation and autonomy does not lead to the adult withdrawing from expressing his views and perspectives but that a safe setting is formed between the participants, where there is room for different perspectives, both agreements and disagreements. This situation between the participants, where both are equal individuals in the work on a common issue, can be related to the psychotherapeutic methodological approach, working alliance (Bordin, 1979), which is about the relationship between therapist and client. This is an understanding of collaboration initially between participants with an uneven power relationship and how the participants, in an experience of alliance and community, activate forces of action and activity to work toward a goal. Bordin (1979) presents the concept with a basis in psychoanalytic theory and how this can be generalized to be an effective factor for many types of collaboration and change processes. His perception of the working alliance is based on a hypothesis that through collaboration one can achieve changes through an agreement on goals, an assignment of task or a series of tasks (agreement on working method), and the development of a bond. This working alliance is not therapeutic in itself, but the collaborative aspect of the relationship between the partners (here linked to an unequal power relationship with a vulnerable child and an expert) affects the effect of the work (Bordin, 1979). Bordin also generalizes this beyond the therapy room and believes that it is also relevant between students and teachers, between community action groups and leaders, and, with some adaptation, also between parents and children. Through working together on common goals, a collaborative alliance can be formed and neutralize some of the power imbalance that can be linked to the context and the various participants’ roles. In this collaboration, both participants (adult and child) have the experience that they complement each other – that it is a mutual dyadic relationship that benefits from each other’s strengths and reflections.

## The four dimensions of child participation

With the preceding theoretical basis for conceptualizing child participation, it is further argued for four parametric dimensions to promote empowerment as a prerequisite for active participation in participation situations. These dimensions are *information*, *autonomy*, *recognition* and *alliance*.

### Dimension A: information – experience of coherence

The need for information is promoted as a key dimension for creating conditions for the child to engage actively in the process. In a general comment from the UN Convention on the Rights of the Child, it is mentioned that although the right to adequate information is not mentioned in the text of the convention, this is a prerequisite for the realization of the right to express oneself (General Comment No. 12 section 25, UN Committee on the Rights of the Child, 2009). Information is thus a crucial component for the child to be able to make clear decisions and to be able to express themself freely in the matter in question. In addition to the rights perspective, the need for information is central to create a framework and space for the child to develop the prerequisites for advancing his views. Information and an overview of their own situation in a larger context will contribute, as Wyness (2006) describes, to the room for activity so that children can act. This will involve knowing the theme, aim, and purpose of the participation and one’s own role in the meeting with the adult. It will also, however, involve a certain amount of information and knowledge about the context, working methods, and opportunities to be able to feel that one has the competence to carry out the actions that are expected of one. Experience of competence (Deci and Ryan, 1985, 2000) is a basic need for activating energy and action so that the child becomes an active participant instead of a pawn in an adult-controlled context. A sense of competence and understanding of the scope of possibilities in the situation can stimulate the child’s experience of agency (Bandura, 1994a) and thus promote the motivation to actively take control of the situation. Such an agentic role is also connected with Antonovsky’s concept of SOC and the experience of the situation as understandable and predictable. Through information and knowledge about the context and their own role in the situation, the child can be helped to experience the situation as clear and meaningful and can thus experience a certain form of control. This is not about being able to control the situation itself but is about understanding and experiencing what is happening as meaningful consequences of action chains. This can thus provide the opportunity to adapt one’s own actions and reactions to the situation and surroundings and be a driver of protecting one’s own interests. Information about the case and the context can thus contribute to making an unfamiliar situation safer and more manageable so that the child’s vulnerability and potential sense of powerlessness in the face of the judicial and administrative procedures are reduced. In this manner, prerequisites are established for the child to get involved and take the initiative regarding activity. These become important perspectives when we come to understanding what promotes action resources in the child in the face of unknown and unsure situations. Information and an overview of the situation in which one takes part is therefore a decisive factor in children taking on an active and constructive role in their own situation, activating forces of agency and protecting their interests. This has to do with the sense of being an active participant in the situation as opposed to being passive and dependent on others. Information is therefore a crucial component for the child to be able to make clear decisions and to form their views in the matter.

The extent to which children are included in matters that concern them may depend on the adult’s understanding of the matter’s degree of burden for the child, and can often lead to adults withholding information about the case to spare the child. For the child to be able to take ownership of his own case, it is still important to be open about the issue and the room for action in which he can participate. Even if the adults invite to take part in a collaboration process, it is not always that the child is receptive to this and wants to participate in a democratic process. This is a choice that the child must make based on their wishes and needs, but adults must be aware that reluctance to participate in participation situations can come from uncertainty in the expectations of what this entails and uncertainty in relation to the child’s role in the collaboration with the adult. It therefore becomes a task for the adult to facilitate processes that are as open as possible and to present the case so that the child can contribute at the level he wishes. It will thus be important to ensure that the child understands the context (the purpose and goal of the meeting), seek a common understanding of the issue and clarify the framework and room for action for working with the issue. This is an important principle and prerequisite whether it applies to everyday contexts, school situations or childcare. It is crucial in every situation that both the adult and the child feel that there is a real need for the children’s participation in the matter (Olsen, 2022), but the child should also feel that he has ownership of the matter in order to mobilize a commitment and his own powers of action in the work.

### Dimension B: autonomy – space to form your views and express yourself

In Article 12 of the Convention on the Rights of the Child, children’s right to “freely express their views” is promoted, particularly in any legal and administrative proceedings concerning the child. This formulation emphasizes that the child should be able to express themself independently of other people’s opinions, wishes, and needs, but it also considers that the child has their own clear views, which they can convey. Article 5 of the UNCRC also makes adults responsible for creating conditions for the child so that they can exercise their rights. As Heffner (1988) understands empowerment, this means not only giving the young people the opportunity to participate and express their opinion but to create frameworks and structures that support the children’s experience of mastery and to develop the ability to express themselves. The children’s experience of empowerment in the situation therefore becomes more important for their active participation than the adult’s inviting participation. Provision must be made for the children’s freedom to express themselves. This can be considered the psychological form of empowerment (Rissel, 1994), which promotes the child’s inner strengths and self-confidence and strengthens the child’s prerequisites for asserting themselves. This is an important component to counteract the experience of powerlessness and lack of influence. It requires both an awareness of how power inequalities affect the child’s experience of freedom to express their own views, as well as time and space to develop and formulate their own opinions. In this aspect, the adults’ facilitation of the child’s experience of autonomy is emphasized as well as how they can support the children’s experience of integrity and self-regulated action in this context. Through this, the child can experience a greater degree of control to take the initiative for their own action; thus, autonomy becomes an important factor in freeing the children’s forces of action. This can facilitate children expressing their own autonomous views on the matter. Through self-determination theory (Deci and Ryan, 2000), emphasis is also placed on the experience of competence to be able to contribute actively to the process, which means that the child must be given time to reflect and shape their opinions before they are expressed in the interaction with the adult. Facilitating a safe space between the adult and the child will also enable the creation of a framework for safe participation so that the child feels strengthened in the situation and motivated to promote his own views. The child’s sense of autonomy and freedom to express themself can in this approach activate the child’s forces of action and resources, and therefore the child’s autonomous views and reflections are promoted.

In practice, this means meeting the child with a belief in his competence and self-worth, and by conveying a genuine interest and need for the child’s contribution, giving the child an independent role in the collaboration. Both in an everyday context, a meeting between the child and the school or in child care, the child needs to sense that the adult searches the child’s own views on the situation, not necessarily to be faced with different options, but through joint reflections on the topic, enough time to think about the case’s issues and room for action, and a safe environment through the experience of relatedness with the adult can free their own views, wishes and needs from perceived expectations and demands in the situation. This means that the adult must make room for the child both feels that he has time to form his own views on the matter, and at the same time feels that it is safe to express them. This issue is explored in an article by Olsen et al. (2022), where the child’s experience of active participation in a collaborative situation with adults is explored and analyzed in the light of Deci and Ryan’s (1985, 2000) self-determination theory. Lundy and McEvoy (2012) also highlight the children’s need for support to formulate their own thoughts and views on the topic, so that we can talk about real prerequisites for participation.

### Dimension C: recognition – the need to feel heard and be taken seriously

An experience of being included in participation processes also depends on a sense of being heard and taken seriously. Lundy (2007) embraces this dimension in “*audience”* and describes how meaningful participation is linked to the children’s need to talk to a significant adult with the power to act. The Convention on the Rights of the Child is clear that the child’s views must be given due weight both in Article 12 and Article 3, where the child’s best interests are the focus. The experience of being heard and taken seriously can thus traditionally be understood as the child helping to determine the outcome of the participation process and the child’s wishes being fulfilled. An empowerment understanding of facilitating child participation, on the other hand, emphasizes the sense of recognition from the adult, and an interest in understanding the child’s situation, perspectives, and needs. Through the sense of the adult’s recognition, respect and acceptance of the child’s situation can be conveyed. Recognition as an empowerment element to promote active and real participation will thus concern the experience of dignity and respect in meeting the adult in the authoritative role and an experience of having a significant value. As Graham and Fitzgerald (2008) point out, an appreciative relationship can lead to increased self-confidence and experience of mastery, and hence to the confidence to be able to convey one’s own views.

An appreciative attitude in meeting the child will also be an important basis for a democratic understanding of the participation process and a step that facilitates openness for everyone’s views and contributions. Based on Schibbye’s (1988, 2009) view, recognition will contribute to the child strengthening their independence and to their identification of need. This helps to create a sense of equality between the adult and the child in the situation, which can thus help to equalize the power imbalances that can reduce the child’s sense of empowerment and active participation in the process. Without an attitude of recognition, there is a risk that the child will have challenges in breaking away from the adults’ expectations and views, thereby reducing the child’s autonomous voice. Recognition therefore becomes important to convey confidence, respect, and equality, which invites the child to promote their own thoughts, reflections, and views on a matter.

In practical implementation, this approach means that the child’s views do not necessarily become decisive in the decision-making process. It is rather the feeling that the adult has understood the child’s views and perspectives, and based on that assesses the child’s best interests. In meeting with the child, the appreciative processes are about listening to what the child has to say, following up on the child’s statements, being open and understanding of the child’s perspectives, feelings, wishes and needs That also includes avoiding correcting or defend the child’s statements, but to search for the premise for the statement. Acknowledgment of the child’s wishes and needs does not mean that these are granted, but it helps ensure that the child’s views can be taken seriously, without this being at the expense of the child’s safety. Studies have also shown that the children themselves do not necessarily want to decide more in participation situations, but they want to be heard and considered in decision-making. The children wish to express their opinion, but still want the adults to be responsible for their best interests.

### Dimension D: alliance – the power of collaboration

Children usually contribute to matters that are initiated and defined by adults and therefore have a role that is not initially characterized by equality. Invitations to participate should therefore consider this skewed distribution of power and facilitate children’s sense of empowerment and the opportunity to influence the situation. In the empowerment model described in this article, the perspective shifts from real participation in the matter and instead highlights the interrelational process and the child’s sense of participating. Less emphasis is thus placed on the children’s contribution to the outcome, and more emphasis is given to how the child helps to influence the process. The child’s ability to assert himself in the interaction will thus be an important component in being able to adopt an active role in the participation process. The collaboration dimension in the empowerment model therefore emphasizes the relational understanding of autonomy presented by Solberg (2022), where the child’s sense of self-worth will promote assertiveness and active involvement. A collaborative dimension of child participation emphasizes that both the child and the adult have meaningful roles that can contribute in their own way to the collaboration process. Such an understanding of collaboration implies not only an acceptance of, but also a necessity for, different points of view and perspectives. Through dynamic forces of autonomy, the matter will then take form according to the initiatives and needs of all participants, and the child will become an integral part of the process. Bordin’s (1979) understanding of working alliance relates to this through the understanding of common goals, agreement on the assignment, and the experience of bonds or relatedness in the work. A common understanding and commitment to the “assignment” can create a sense of equality that highlights the value of the child’s voice. The sense of being an important element that brings something useful to the collaboration with the adult can also affect the dynamic in which adults and children complement each other with their perspectives and points of view. This highlights the value of the child’s participation, as there is a use for the resources that the child can contribute. Emphasis on collaboration can thus contribute to the sense of self-worth in meeting the adult and promote a sense of empowerment. This can free the children’s agency to become active participants in the process and support real participation.

The work of Olsen (forthcoming)^1^ illustrates this dyadic relation as cogwheels in a participatory machinery, where each participants contribute with their own significant functions for the collaboration. In this manner, the participants depend on each other, and the effect of the collaboration would have been difficult to generate alone. The question of how much the children alone contribute to the collaboration then becomes less important, but the joint process that both children and adults go through is emphasized. Such a process involves mutual orientation, inspiration, understanding and influence. In a qualitative dyadic process, adults can facilitate the children’s contribution to the collaboration to be more targeted and meaningful.

In practice, it’s about a sense of being on the same team and “pulling in the same direction.” It is perhaps more natural to enter good collaborative alliances in everyday contexts and within the family, while in relationships where there is a professional distance between, for example, teacher and student or child and counselor, it requires more conscious processes to equalize the power relations and liberate forces of agency. These processes are described in Olsen (2022) and emphasize the children’s sense of value, a safe social environment, inclusion through information and an exploratory working method.

## Final reflections

This article refers to a theoretical approach to the inclusion of children in participation processes through an empowerment perspective. Through theories taken from the field of social and educational psychology, it is argued that adults can facilitate ethically sound participation processes that consider the child’s vulnerable position, both when dealing with adults in judicial and administrative procedures, but also in more everyday situations at school and at home. The model includes a methodological idea that is built on basic viewpoints of respect and equality in encounters between people, and therefore the children’s age, abilities and expertise is not problematized or thematized in this session. Reference is nevertheless made to the children’s age and maturity as guiding considerations, not from a development perspective that focuses on maturity and lack of competence but based on an alternative construction of children with an understanding that we have different competences (Solberg, 1996; Christensen and Prout, 2002). The empowerment model for participation relies on an understanding of people’s basic psychological needs in the face of unknown and vulnerable situations and how one can gain a sense of coping and action. The basic viewpoint is that children are a vulnerable group in general when faced with adults in positions of authority, and therefore the power dimension is a central element in understanding children’s opportunities and prerequisites for active and real participation.

The four dimensions of the model, *information, autonomy, recognition*, and *alliance* are presented as pillars for the active and real inclusion of children in participation processes. The main essence of this conceptual understanding of child participation is that it is based on a collaborative approach where the children take an active role in working with the issue, as opposed to being a passive piece where adults manage and have control. The idea is that through being included in the aim and purpose of the matter, and at the same time being part of the process of exploring solutions and measures, the child will be encourage to a sense of strength and action (empowerment) and supported to take an active role in the participation process together with the adult. This could contribute to reducing the sense of powerlessness and passive and tokenistic forms of participation and to promoting the children’s inner strengths and protection forces in the face of unfamiliar and stressful situations. In this paper, real participation is not understood as the outcome and result of the decision-making process. The model presents a theoretical understanding of child participation which emphasizes facilitating the child’s agency as a methodological approach to assuming an active role in interactions with adults. Real participation will thus be about the child’s sense of empowerment and the opportunity to express their own views free of the adults’ expectations or contextual pressure. This will be the facilitation of an ethically justifiable practice that considers the uneven power relations between child and adult in the process. The main essence of this conceptual understanding of child participation is that it is based on a collaborative approach where the children take an active role in working with the issue, as opposed to being a passive piece where adults manage and have control. The idea is that through being included in the aim and purpose of the matter, and at the same time being part of the process of exploring solutions and measures, then the children will be able to have the prerequisites to achieve real participation.

Based on the general principles of the model that have been tried to be freed from specific context, the model can be used in most participation situations as basic methodological principles. For example, in classroom situations and teacher-pupil relationships, it will be about having common goals as a starting point. Information must then be linked to frameworks and room for action and a common understanding of the meaning and purpose of the activity. Space must be created for the pupils to formulate their opinions through strengthening the pupils’ sense of autonomy and agency – that they have something meaningful to contribute, and the pupils must feel that the teacher both understands their wishes and needs but also takes these into account in the assessment of what is ultimately best for the students. The experience of an alliance between teacher and students is the last element that will increase motivation and active participation for the students, and even though the child and adult in such a relationship have evened out some of the skewed power relations, this will not come at the expense of the children’s safety in the decision making. This is because the task is goal-and framework-driven, and the adult still has a leadership role and is tasked with promoting the child’s best interests. In this assessment, provision is made for the children to identify and communicate their wishes and needs, and these are considered in the decision-making situation. The model is two-way and allows both for adults to help and support children in their assessment and formulation of their contribution in the collaboration, but also for the children, through increased autonomy and experience of empowerment, to influence the adults’ decisions. This dynamic collaborative approach moves away from information transfer (one way or the other – see model in article Olsen, 2022) and allows for both parties to help develop and design a “solution” to the problem.

The model does not absolve adults from their responsibilities in their professional and educational role; rather, it makes clear how the responsibility for constructing contexts of participation rests primarily with adults, who have greater power in a relationship that remains asymmetrical. It is important to explicitly clarify that the model does not discuss the children’s degree of decision-making authority but highlights how participation can be understood as a collaborative process in which the children must be included. The adults retain their decision-making authority, but by entering a cooperative alliance with the child, with the problem itself (small or large) as a starting point, the adult will have greater basis for making decisions that are in the best interests of the child. This not only includes adults being more sensitive to the children’s wishes and needs and taking this seriously, but to an equal extent this is about adults communicating their needs to the children in such a way that the children can take this into account as part of the interaction. Only in this manner can we talk about meaningful collaborative relationships that are based on mutual respect for each other’s participation and contribution. Each participant (both adults and children) has different needs that must be communicated in the work with the issue of the case. As described in Olsen (2022) and Olsen et al. (2022), it is of great importance for children’s mobilization of agency and forces of participation that the adult’s role is adjusted from “the one who knows and can do everything” to a co-player who has a real need for the child’s contribution to the matter. In this understanding, realizing that adults’ safeguarding of the child’s best interests [art 1, United Nations Convention on the Rights of the Child (UNCRC), 1989] is dependent on the child’s mediation and advocacy for their wishes and needs, and the adult’s role in enabling the child’s real and meaningful participation.

## Possible limitations of the work and suggestions for further research

The study of the conceptual significance of child participation has relied on a pragmatic approach (Dewey, 1925), where truth is set in context with practice and the empirical world to have meaning. The theoretical model in this paper is thus presented as an idea, and must be introduced, tested, and discussed in the face of empirical reality and the relevant issues in order to find its instrumental value. However, it is impossible to capture the entire complexity of the phenomenon in a theory, and therefore some parameters have been selected which together form a simplified and abstract model of the phenomenon and reality. The focus is therefore on common features, theoretical character and examples, and not specification. A clear definition and presentation of a procedure can provide a stable finding, while an isolated empirical content will be difficult to transfer to other given instances, as the transfer value will depend on context. A sensitizing concept will, in contrast to a clear definition and fixed characteristics, refer more to a direction to look for and refer to a feeling in the approach to the empirical. The purpose of approaching the concept with such vagueness is that it can help direct the focus in a particular direction – not present a conclusion or a particular procedure for practice.

As a further step in this knowledge development, more attention should be paid to testing the model’s relevance and reliability in several contextual contexts to further develop and create greater social scientific weight. Further research should thus direct more attention to the actual operationalization and implementation of the model linked to empirical consequences. The model can, among other things, be used as an analysis tool for observations of participation practices or collaborative practices in several different contexts, for example forms of collaboration such as interdisciplinary collaboration meetings, collaboration between support services and the inclusion of parental collaboration. This type of research can help to further develop and create greater weight in the theoretical argument. Both qualitative analysis/observation studies and intervention studies can provide an important supply of knowledge that strengthens the empirical relevance. In addition, an operationalization with a view to quantitative and psychometric measurements of children’s experience of participation will also provide a valuable knowledge contribution to implementation strategies, as well as recording the qualitative participation practice in the field of practice today. A validation of such a tool can contribute to easier access to research-based knowledge for the field of practice, and that empirical knowledge in the field can be more easily transferred to an academic and theoretical level. The study can thus contribute to strengthening the bridge of knowledge between theory and practice and be a significant contribution to the development of social science, meaning in this context how the idealistic intention of child participation can be realized in practice.

## Data availability statement

The original contributions presented in the study are included in the article/supplementary material, further inquiries can be directed to the corresponding author.

## Author contributions

The author confirms being the sole contributor of this work and has approved it for publication.

## Footnotes

^1^Olsen, R. K. (forthcoming). The value of child participation in research – a qualitative child-centered approach to the early development of an empowerment inventory for children.

## References

[ref1] Alvesson M. Sköldberg K. (2009). Reflexive methodology: New vistas for qualitative research. 2nd Edn. Thousand Oaks, CA: SAGE.

[ref2] Antonovsky A. (1987) Unraveling the mystery of health: How people manage stress and stay well. San Francisco: Jossey-Bass.

[ref4] Archard D. Skivenes M. (2009). Hearing the child. Child Fam. Soc. Work 14, 391–399. doi: 10.1111/j.1365-2206.2008.00606.x

[ref5] Aronsson K. Hendegaard M. Højholt M. Ulvik O. S. (2018) Children, childhood, and everyday life: children’s perspectives. Charlotte, NC: Information Age Publishing, Inc.

[ref6] Askheim O. P. (2017). Brukermedvirkningsdiskurser i den norske velferdspolitikken. Tidsskrift for velferdsforskning 20, 134–149. doi: 10.18261/issn.2464-3076-2017-02-03

[ref7] Bae B. (2010). Realizing children’s right to participation in early childhood settings: some critical issues in a Norwegian context. Early Years 30, 205–218. doi: 10.1080/09575146.2010.506598

[ref8] Bandura A. (1986) Social foundations of thought and action: A social cognitive theory (prentice hall series in social learning theory). Englewood Cliffs, NJ: Prentice-Hall.

[ref9] Bandura A. (1994a). “Self-efficacy” in Encyclopedia of human behavior. ed. Ramachaudran V. S., vol. 4 (New York: Academic press), 71–81. reprinted in H. Friedman [ed.] [1998] Encyclopedia of mental health. San Diego: Academic Press

[ref10] Bandura A. (1994b). “Social cognitive theory and exercise of control over HIV infection” in Preventing AIDS. AIDS prevention and mental health. eds. DiClemente R. J. Peterson J. L. (Boston: Springer), 25–59.

[ref11] Bennett Woodhouse B. (2003). Enhancing children’s participation in policy formulation. Ariz. Law Rev. 45, 750–763.

[ref12] Blumer H. (1954). What is wrong with social theory. Am. Sociol. Rev. 19, 3–10. doi: 10.2307/2088165

[ref13] Bordin E. S. (1979). The generalizability of the psychoanalytic concept of the working alliance. Psychotherapy 16, 252–260. doi: 10.1037/h0085885

[ref14] Bowen G. A. (2019). “Sensitizing concepts” in Sage research methods foundations. ed. Atkinson P. (London: SAGE Publications Ltd.).

[ref15] Children’s Ombudsman. (2017). Uten mål og mening? Elever med spesialundervisning i grunnskolen. Barneombudets fagrapport. Available at: https://www.barneombudet.no/uploads/documents/Publikasjoner/Fagrapporter/Uten-mal-og-mening.pdf (Accessed June 23, 2023).

[ref16] Christensen P. Prout A. (2002). Working with ethical symmetry in social research with children. Childhood 9, 477–497. doi: 10.1177/0907568202009004007

[ref17] Clark A. Moss P. (2001). Listening to young children: The mosaic approach. London: National, Children’s Bureau for the Joseph Rowntree Foundation.

[ref18] Deci E. L. Ryan R. M. (1985). The general causality orientations scale: self-determination in personality. J. Res. Pers. 19, 109–134. doi: 10.1016/0092-6566(85)90023-6

[ref19] Deci E. L. Ryan R. M. (2000). The “what” and “why” of goal pursuits: human needs and the self-determination of behavior. Psychol. Inq. 11, 227–268. doi: 10.1207/S15327965PLI1104_01

[ref20] Department of Children and Youth Affairs (DCYA). (2015) National strategy on child and youth participation. Dublin: DCYA.

[ref9009] Dewey J. (1925). The meaning of value. J Philos, 22, 126–133. doi: 10.2307/2014306

[ref21] Ergler C. R. (2017). Advocating for a more relational and dynamic model of participation for child researchers. Social Inclusion 5, 240–250. doi: 10.17645/si.v5i3.966

[ref22] Flemmen A. B. (2017). “Sensitizing concepts in action: expanding the framework” in Concepts in action. Eds. Leiulfsrud H. Sohlberg P. (Boston: Brill), 79–94.

[ref23] Graham A. Fitzgerald R. M. (2008). “Young People Big Voice’: reflections on the participation of children and young people in a university setting,” in Involving children and young people in research: a compendium of papers and reflections from a think tank co-hosted by the Australian Research Alliance for Children and Youth and the NSW Commission for Children and Young People on 11 November 2008. Australian Research Alliance for Children and Youth. New South Wales Commission for Children and Young People, 64–75.

[ref24] Graham A. Fitzgerald R. (2010). Progressing children’s participation: exploring the potential of a dialogical turn. Childhood 17, 343–359. doi: 10.1177/0907568210369219

[ref25] Green L. W. George M. A. Daniel M. Frankish C. J. Herbert C. P. Bowie W. R.. (1995). Study of Participatory Research in Health Promotion. Ottawa, Ontario: Royal Society of Canada.

[ref26] Hagquist C. Starrin B. (1997). Health education in schools—from information to empowerment models. Health Promot. Int. 12, 225–232. doi: 10.1093/heapro/12.3.225

[ref27] Hart R. A. (1992) ‘Children’s participation: From tokenism to citizenship. No. inness92/6. Florence: Innocenti Essay.

[ref28] Heffner K. (1988). The evolution of youth empowerment at a youth newspaper. Soc. Policy 19, 21–24.

[ref29] Hegel G. W. F. (1999) Hegel: Political writings. Cambridge: Cambridge University Press.

[ref9010] Holzkamp K. (2015). “Conduct of everyday life as a basic concept of critical psychology”, in Psychology and the conduct of everyday life. Eds. Schraube E. Højholt C. (New York, NY: Routledge). 65–98.

[ref30] Kant I. (1964) Groundwork of the metaphysics of morals. Paton H. J.. New York: Harper Torchbooks.

[ref31] Kellett M. (2011). Empowering children and young people as researchers: overcoming barriers and building capacity. Child Indic. Res. 4, 205–219. doi: 10.1007/s12187-010-9103-1

[ref32] Kilkelly U. Kilpatrick R. Lundy L. Moore L. Scraton P. Davey C.. (2005) Children’s rights in Northern Ireland. Belfast: Northern Ireland Commissioner for Children and Young People.

[ref33] Kvernbekk T. (2002). “Vitenskapsteoretiske perspektiver” in Innføring I forskningsmetodologi. ed. Lund T. (Oslo: Unipub), 19–78.

[ref9020] Leeson C. (2007). My life in care: experiences of non‐participation in decision‐making processes. Child & Family Social Work, 12, 268–277. doi: 10.1111/j.1365-2206.2007.00499.x

[ref34] Lúcio J. I’Anson J. (2015). Children as members of a community: citizenship, participation and educational development – an introduction to the special issue. European Educ. Res. J. 14, 129–137. doi: 10.1177/1474904115571794

[ref35] Lundy L. (2007). Voice’ is not enough: Conceptualising article 12 of the United Nations convention on the rights of the child. Br. Educ. Res. J. 33, 927–942. doi: 10.1080/01411920701657033

[ref36] Lundy L. McEvoy L. (2012). Children’s rights and research processes: assisting children to (in) formed views. Childhood (Copenhagen, Denmark) 19, 129–144. doi: 10.1177/0907568211409078

[ref37] Mead G. H. (1934) Mind, self, and society. Chicago: University of Chicago Press.

[ref38] Morrow V. (1999). We are people too: children’s and young people’s perspectives on children’s rights and decision-making in England. Int. J. Child. Rights 7, 149–170. doi: 10.1163/15718189920494318

[ref39] NOU 2020:14. (2020). Ny barnelov – Til barnets beste. Barne-og familiedepartementet. Available at: https://www.regjeringen.no/no/documenter/nou-2020-14/id2788399/ (Accessed June 23, 2023).

[ref40] Ochs E. Izquierdo C. (2009). Responsibility in childhood: three developmental trajectories. Ethos 37, 391–413. doi: 10.1111/j.1548-1352.2009.01066.x

[ref41] Olsen R. K. (2022). “Now I understand why she needs our help”: a qualitative case study on collaborative alliances with children in research. Int. J. Child. Rights 30, 990–1020. doi: 10.1163/15718182-30040007

[ref43] Olsen R. K. Stenseng F. Kvello Ø. (2022). Key factors in facilitating collaborative research with children: a self-determination approach. Academic Quarter 24, 135–148. doi: 10.54337/academicquarter.vi24.7256

[ref44] Prilleltensky I. (2001). Value-based praxis in community psychology: moving toward social justice and social action. Am. J. Community Psychol. 29, 747–778. doi: 10.1023/A:1010417201918 11594698

[ref45] Prilleltensky I. (2010). Child wellness and social inclusion: values for action. Am. J. Community Psychol. 46, 238–249. doi: 10.1007/s10464-010-9318-9 20532614

[ref46] Rappaport J. (1981). In praise of paradox: a social policy of empowerment over prevention. Am. J. Community Psychol. 9, 1–25. doi: 10.1007/BF00896357, PMID: 7223726

[ref47] Rappaport J. (1984). Seeking justice in the real world: a further explication of value contexts. J. Community Psychol. 12, 208–216. doi: 10.1002/1520-6629(198407)12:3<208::AID-JCOP2290120304>3.0.CO;2-#, PMID: 10267398

[ref48] Rissel C. (1994). Empowerment: the holy grail of health promotion? Health Promot. Int. 9, 39–47. doi: 10.1093/heapro/9.1.39

[ref49] Ryan R. M. (1993). “Agency and organization: intrinsic motivation, autonomy and the self in psychological development” in Nebraska symposium on motivation: Developmental perspectives on motivation. ed. Jacobs J., vol. 40 (Lincoln: University of Nebraska Press), 1–56. 1340519

[ref50] Saglietti M. Zucchermaglio C. (2022). Children’s participation and agency in Italian residential care for children: adult-child interactions at dinnertime. Eur. J. Psychol. Educ. 37, 55–83. doi: 10.1007/s10212-021-00531-7

[ref51] Save the Children USA. Save the Children US Annual Report 2003. (2004) Fairfield, CT: Save the children USA.

[ref52] Schibbye A. L. L. (1988) Familien: Tvang og mulighet. Om samspill og behandling. Oslo: Universitetsforlaget.

[ref53] Schibbye A. L. L. (2009) Relasjoner: et dialektisk perspektiv på eksistensiell og psykodynamisk psykoterapi. Oslo: Universitetsforlaget.

[ref54] Shier H. (2001). Pathways to participation: openings, opportunities and obligations: a new model for enhancing children’s participation in decision-making, in line with article 12.1 of the United Nations convention on the rights of the child. Child. Soc. 15, 107–117. doi: 10.1002/CHL617

[ref55] Sinclair R. Cronin K. Lanyon C. Stone V. Hulusi A. (2002). Aim high stay real: Outcomes for children and young people: The views of children, parents and practitioners (London: Children and Young People’s Unit).

[ref56] Skauge B. Storhaug A. S. Marthinsen E. (2021). The what, why and how of child participation—a review of the conceptualization of “child participation” in child welfare. Soc. Sci. 10:54. doi: 10.3390/socsci10020054

[ref57] Smith R. Monaghan M. Broad B. (2002). Involving young people as co-researchers: facing up to the methodological issues. Qual. Soc. Work. 1, 191–207. doi: 10.1177/1473325002001002619

[ref58] Solberg A. (1996). “The challenge in child research: from “being” to “doing”” in Children in families: Research and policy. eds. Brannen J. O’Brien M. (London: Falmer).

[ref59] Solberg B. (2020). Pasientautonomi og brukermedvirkning i helsetjenesten – hvorfor så verdifullt? Michael, 17, 45–55.

[ref60] Solberg B. (2022). Samtykkekompetanse og den (u)frie vilje. Available at: https://youtu.be/gjnb8VSXoTg (Accessed January 13, 2017).

[ref61] Sue D. W. (1981) Counselling the culturally different. New York: John Wiley & Sons.

[ref62] Suppe F. (1989). The semantic conception of theories and scientific realism. Champaign, IL: University of Illinois Press.

[ref63] Suppe F. (2000). Understanding scientific theories: an assessment of developments, 1969-1998. Philos. Sci. 67, S102–S115. doi: 10.1086/392812

[ref64] Szoko N. Dwarakanath N. Miller E. Chugani C. D. Culyba A. J. (2022). Psychological empowerment and future orientation among adolescents in a youth participatory action research program. J. Community Psychol. 51, 1851–1859. doi: 10.1002/jcop.22935, PMID: 36095077 PMC10008464

[ref65] Thomas N. (2007). Towards a theory of Children’s participation. Int. J. Child. Rights 15, 199–218. doi: 10.1163/092755607X206489

[ref66] Thomas N. O’Kane C. (1998). The ethics of participatory research with children. Child. Soc. 12, 336–348. doi: 10.1111/j.1099-0860.1998.tb00090.x

[ref67] Treseder P. (1997) Empowering children and young people. London: Save the Children.

[ref68] Ulset G. Thoresen S. H. Thaulow K. Melby L. Bruteig R. Paulsen V. (2023). Barn og unges medvirkning i spesialiserte hjelpetiltak. Available at: https://www.researchgate.net/profile/Veronika-Paulsen-2/publication/368882114_Barn_og_unges_medvirkning_i_spesialiserte_hjelpetiltak/links/63ff4047574950594553fde6/Barn-og-unges-medvirkning-i-spesialiserte-hjelpetiltak.pdf (Accessed June 23, 2023).

[ref69] UN Committee on the Rights of the Child. (2009) General comment no.12 the right of the child to be heard. Geneva: UN Committee on the Rights of the Child.

[ref70] United Nations Convention on the Rights of the Child (UNCRC) (1989). Assembly, U. G. United Nations Treaty Series 1577, 1–23.

[ref71] Van Bijleveld G. G. Dedding C. W. Bunders-Aelen J. F. (2015). Children’s and young people’s participation within child welfare and child protection services: a state-of-the-art review. Child Fam. Soc. Work 20, 129–138. doi: 10.1111/cfs.12082

[ref72] Van Fraassen B. C. (1980). The scientific image. Oxford: Oxford University Press.

[ref73] Vis S. A. Christiansen Ø. Havnen K. J. S. Lauritzen C. Iversen A. C. Tjelflaat T. (2020). Barnevernets undersøkelsesarbeid-fra bekymring til beslutning. Samlede resultater og anbefalinger. Tromsø: Uit Norges Arktiske Universitet.

[ref74] Viviers A. (2010) The ethics of child participation. Doctoral dissertation. Pretoria: University of Pretoria.

[ref75] Viviers A. Lombard A. (2013). The ethics of children’s participation: fundamental to children’s rights realization in Africa. Int. Soc. Work. 56, 7–21. doi: 10.1177/0020872812459066

[ref9030] Wong N. T. Zimmerman M. A. Parker E. A. (2010). A typology of youth participation and empowerment for child and adolescent health promotion. American Journal of Community Psychology, 46, 100–114. doi: 10.1007/s10464-010-9330-0 20549334

[ref76] Woolfson R. C. Heffernan E. Paul M. Brown M. (2010). Young people’s views of the child protection system in Scotland. Br. J. Soc. Work 40, 2069–2085. doi: 10.1093/bjsw/bcp120

[ref77] Wyness M. (2006). Children, young people and civic participation: regulation and local diversity. Educ. Rev. 58, 209–218. doi: 10.1080/00131910600584173

